# Protein SUMOylation modification and its associations with disease

**DOI:** 10.1098/rsob.170167

**Published:** 2017-10-11

**Authors:** Yanfang Yang, Yu He, Xixi Wang, Ziwei liang, Gu He, Peng Zhang, Hongxia Zhu, Ningzhi Xu, Shufang Liang

**Affiliations:** 1 State Key Laboratory of Biotherapy and Cancer Center, West China Hospital, Sichuan University, and Collaborative Innovation Center for Biotherapy, No.17, 3rd Section of People's South Road, Chengdu, 610041, People's Republic of China; 2 Department of Urinary Surgery, West China Hospital, Sichuan University, Chengdu, 610041, Sichuan, People's Republic of China; 3 Laboratory of Cell and Molecular Biology & State Key Laboratory of Molecular Oncology, Cancer Institute & Cancer Hospital, Chinese Academy of Medical Sciences, Beijing, 100034, People's Republic of China

**Keywords:** SUMOylation, SUMO pathway, deSUMOylation, disease

## Abstract

SUMOylation, as a post-translational modification, plays essential roles in various biological functions including cell growth, migration, cellular responses to stress and tumorigenesis. The imbalance of SUMOylation and deSUMOylation has been associated with the occurrence and progression of various diseases. Herein, we summarize and discuss the signal crosstalk between SUMOylation and ubiquitination of proteins, protein SUMOylation relations with several diseases, and the identification approaches for SUMOylation site. With the continuous development of bioinformatics and mass spectrometry, several accurate and high-throughput methods have been implemented to explore small ubiquitin-like modifier-modified substrates and sites, which is helpful for deciphering protein SUMOylation-mediated molecular mechanisms of disease.

## 1. Introduction

Protein post-translational modifications (PTMs) include phosphorylation, glycosylation, acetylation, ubiquitination, SUMOylation and many others [1]. As a competitor of ubiquitination, protein SUMOylation has become one of research hotspots in recent years. SUMOylation is one of PTMs, in which a member of the small ubiquitin-like modifier (SUMO) family of proteins is conjugated to lysine (Lys) residues in target proteins. SUMOylation modification is reversible and dynamic process, in which the modified proteins can be deSUMOylated by sentrin/SUMO-specific proteases (SENPs) [2]. The reversible attachment of a SUMO to a protein is controlled by an enzymatic pathway that is analogous to the ubiquitination pathway [3].

More and more researches have realized the importance of SUMOylation in the normal function of the body [4]. Along with the accumulating knowledge on its biological functions, SUMOylation has been reported to regulate protein subcellular localization, protein–DNA binding, protein–protein interactions, transcriptional regulation, DNA repair and genome organization [5]. Moreover, there is abundant evidence to show the aberrance of SUMO regulation is highly associated with various diseases, including cardiac disease [6], neurodegenerative disease [7] and cancers [8].

## 2. Small ubiquitin-like modifier family members

The SUMO family is a highly conserved PTM form in all eukaryotes, which is required for viability of most eukaryotic cells. There is only one SUMO gene SMT3 in budding yeast, while at least eight SUMO paralogues are present in plants [9]. In mammalian cells, SUMO proteins consist of four components, including SUMO-1, SUMO-2, SUMO-3 and SUMO-4 [3,10]. SUMO-1 is one 101-amino-acid protein with 11.6 kDa. The SUMO-2 shares 95% homology with SUMO-3. The SUMO-2 and SUMO-3 differ from each other by only three N-terminal residues and have yet to be functionally distinguished, but together they share only approximately 45% homology with their paralogue SUMO-1 [11]. Despite the low sequence homologies, SUMO-1 and SUMO-2/3 share very similar three-dimensional structures. SUMO-4 is the least well characterized SUMO isoform. SUMO-4 is probably non-conjugated under physiological conditions. A gene coding for SUMO-4 was identified through analysis of single-nucleotide polymorphisms associated with type 1 diabetes [12]. In addition, the expression of SUMO-4 is increased in pre-eclamptic placentas and in models of oxidative stress and hypoxic injury [13]. Therefore, SUMO-4 may be a potential post-translational mechanism in the stressed pre-eclamptic placenta.

Nevertheless, there are important differences between mammalian SUMO paralogues. First, most of target proteins are modified exclusively by SUMO-1 *in vivo*, which is the dominant SUMO type among the four representative ones in mammalian cells [14]. Some other target proteins are conjugated to SUMO-2/3, or readily conjugated with all SUMO paralogues. SUMO is important for cellular response to stress [15,16], such as heat shock, DNA damage and oxidative stress [5,10]. SUMO-1 and SUMO-2/3 have different dynamics and responses to physiological stresses in mammalian cells. For instance, the nucleoplasmic SUMO-1 is more resistant to bleaching than the SUMO-2 or SUMO-3 in HeLa cells [17]. So cellular SUMO-1 dynamical transitions between SUMOylation and deSUMOylation take more time than the modification dynamical reactions of the SUMO-2 and SUMO-3.

## 3. Signal crosstalk of SUMOylation with ubiquitination

### 3.1. Small ubiquitin-like modifier is similar to ubiquitin in structure

In the aspect of the structure between SUMOs and ubiquitin, although the amino acid sequence alignments exist 18% identical between ubiquitin and SUMO-1, they have the same three-dimensional structure, especially β-sheet wraps a spherical folding of α-helix [18]. In addition, the position of the two C-terminal Gly residues required for isopeptide bond formation is conserved between ubiquitin and SUMO-1 [18,19]. The biological effects of ubiquitination and SUMOylation are both largely determined by the binding of proteins bearing specific interaction domains [20,21]. However, SUMO has an N-terminal extension that is not found in ubiquitin [20], which is probably the key point that SUMOylation has a different cell biological function than ubiquitination.

### 3.2. Biochemical process of SUMOylation and ubiquitination

The biochemical process of protein SUMOylation is related to ubiquitination. Ubiquitin and SUMO, the most prominent members of a conserved protein family of ubiquitin-like proteins (Ubls), can be attached to Lys residues of target proteins via an isopeptide bond [22]. The ubiquitin-like modifications are carried out in a three-step cascade mechanism requiring the consecutive action of activating enzymes (E1s), conjugating enzymes (E2s) and ligases (E3s). In human cells, ubiquitination is mediated by two E1 ubiquitin activating enzymes, approximately 35 kinds of E2 ubiquitin conjugating enzymes and a variety of E3 ubiquitin ligases. The ubiquitinated proteins are recognized by receptors that contain ubiquitin-binding domains, while the deubiquitinases, a specialized family of proteases, remove ubiquitin modifications [23].

**Figure 1.**
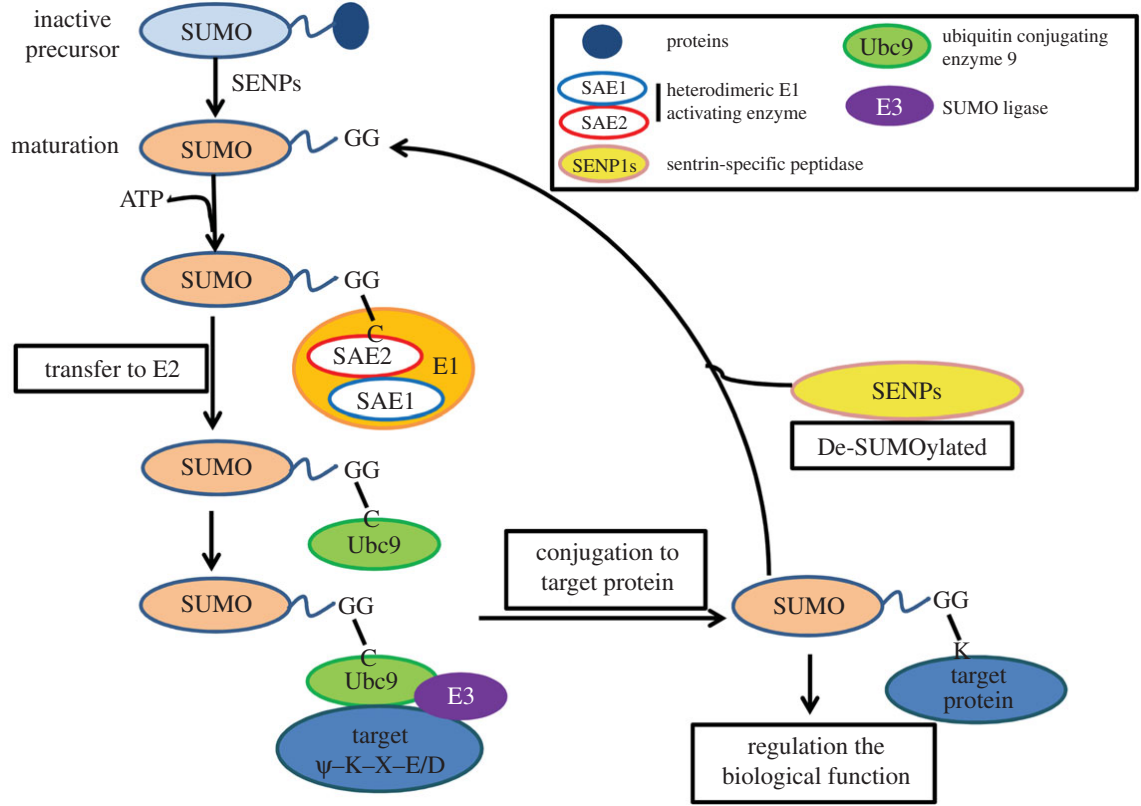
Biochemical process of SUMO modifications in mammal cells. All small ubiquitin-like modifier (SUMO) paralogues are synthesized as pre-proteins that are first cleaved by a SENP to expose a carboxy-terminal diglycine (GG) motif (maturation). An ATP-requiring activation step by the heterodimeric E1activating enzyme (including SAE1 and SAE2) then generates a SUMO–SAE2 thioester. SUMO is then transferred to the E2 conjugating enzyme Ubc9, again forming a thioester. This last step usually requires a SUMO E3 ligase to bring about an isopeptide bond between the SUMO C-terminus and a lysine within the target protein.

**Figure 2.**
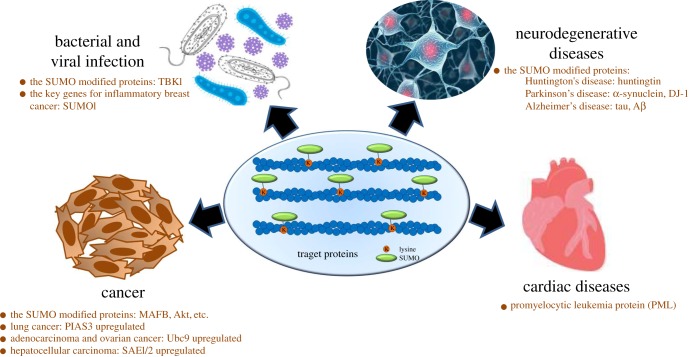
Relationship of SUMO-modified proteins with different diseases, along with some examples of representative proteins and SUMO pathway members.

Similarly, SUMOylation, an analogous modification of ubiquitination, is similar to the conjugation pathway of ubiquitin in the biochemical process (figures 1 and 2), which is performed in turn under the E1, E2 and E3 enzyme catalysis [20]. During protein SUMOlytion, SUMOs are synthesized as propeptides that require cleavage to reveal C-terminal diglycine motifs by SENPs in mammal cells [24]. SUMOs are then activated by an ATP-dependent heterodimer of SUMO activating enzyme subunit 1 (SAE1) and SAE2 [25], which passes the activated SUMO protein onto the specific and unique conjugating enzyme, a ubiquitin conjugating enzyme 9 (Ubc9), through a *trans*-esterification reaction and forming a high-energy thioester bond [26]. The Ubc9 usually acts in conjunction with an E3 ligating enzyme, then catalyses SUMO conjugation to the substrate [27]. Finally, SUMO conjugation forms an isopeptide bond between the SUMO C-terminus and a ε-amino group of a Lys within the target protein [11]. A number of proteins have been discovered to have SUMO E3 activity, including Ran binding protein 2 (RanBP2), the protein inhibitor of activated STAT (PIAS), the polycomb protein Pc2 and others [21], which enhance SUMO conjugation to proteins. While the removal of SUMO modification from a protein is mediated by SENPs [28]. Members involved in SUMO pathway in mammal cells are summarized in table 1. In addition, protein SUMOylation requires a consensus SUMOylation motif in the target protein. For example, although there are several Lys residues in a protein, only a few of them could be true SUMOylation sites. SUMO-1, SUMO-2 and SUMO-3 interact with the same N-terminal region of the E2 conjugating enzyme Ubc9 with similar affinities. In general, many SUMOylation sites follow a consensus motif ψ–K–X–E or ψ–K–X–E/D (ψ is a hydrophobic amino acid, K is the target Lys, X is any amino acid and D/E is Asp or Glu) [21].

**Table 1.** The members of SUMO pathway in mammal cells.

members of SUMO pathway	*Homo sapiens*
SUMO	SUMO-1,-2,-3,-4
activating enzyme E1	SAE1,SAE2
conjugating enzyme E2	Ubc9
ligase E3	RanBP2;PIAS1,-2,-3,-4;Pc2 and etc.
SUMO protease	SENP-1,-2,-3,-4,-5,-6,-7

A growing number of proteins have been reported to act as substrates for both ubiquitination and SUMOylation. The modified proteins have a wide range of functions, which are mainly found in their modified substrates [8]. These two modifications between ubiquitination and SUMOylation have many communications in biological functions, including the control of signal transduction pathways, the maintenance of chromosome integrity and genomic stability.

### 3.3. Correlation between SUMOylation and ubiquitination-mediated biological functions

Although protein SUMOylation and ubiquitination both act on the Lys amino acid residue, sometimes they are cooperated, and other times they are competitively modified for a target protein. SUMO modification usually increases protein stability. For instance, the SUMOylation of Oct4 significantly increased Oct4 stability and its DNA binding ability during embryonic and germ cell development [29]. While SUMO regulates the expression of tripartite motif-containing proteins TRIM21, which functions as the Oct-1 ubiquitin E3 ligase to control Oct-1 degradation. Therefore, a higher TRIM21 expression enhances Oct-1 ubiquitination and reduces Oct-1 stability consequently [30].

Some proteins can be simultaneously modified by SUMO or ubiquitin along with different even opposite roles mediated by each modification. For example, SENP1 plays a key role in the regulation of the hypoxic response through regulation of HIF1α stability. In this regulation process, HIF1α SUMOylation can serve as a direct signal for the ubiquitin-dependent degradation of VHL [31]. Promyelocytic leukaemia protein (PML) could be SUMOylated and ubiquitinated when exposed to arsenic trioxide. A pathogenic fragment of huntingtin (HTT) protein can be modified by both SUMO-1 and ubiquitin at the same Lys residue. The SUMOylation of HTT-fragment increases neurodegeneration, whereas its ubiquitination decreases neurodegeneration in a Huntington's disease model [32]. PES1 is a component of the PeBoW complex; when stimulated by oestrogen, the SUMOylation of PES1 upregulates its stability and function via inhibiting its ubiquitination [33]. Post-translational modification of proliferating cell nuclear antigen PCNA can be modified by ubiquitin and SUMO in response to DNA damage [34].

In conclusion, SUMO modification has been shown to compete with ubiquitination for common Lys residues in most cases. On the other hand, SUMO modification also cooperates with ubiquitination to regulate biochemical function.

## 4. Protein SUMOylation relates to multiple diseases

### 4.1. SUMOylation and cancer

Recently, there are many studies have shown that expression of the SUMO E1 activating enzyme (a heterodimer of SAE1 and SAE2), the SUMO E2 conjugating enzyme (Ubc9) or the SUMO E3 ligases appears to be enhanced in numerous cancers [8,35–37]. The expression level of Ubc9 E2 is upregulated in adenocarcinoma and ovarian cancer cells, and PIAS3 is also increased with different degrees in lung cancer, breast cancer, prostate cancer and colorectal cancer [8]. The enzymes involved in SUMO modification is usually increased, which is closely related to the pathogenesis of hepatocellular carcinoma (HCC). For example, the expression of SAE1/2 is significantly upregulated in cancer tissues of HCC patients [38]. Survival rate of patients with liver cancer is related to the expression level of SUMO-2. The only E2 enzyme Ubc9 is overexpressed in HCC during SUMO modification [39], while SENP2, which regulates the process of removing SUMO modification, can inhibit the proliferation of HCC cells [40,41]. Moreover, SUMOylation is important in the development of multidrug resistance in HCC [42].

In addition, human tumorigenesis is closely related to SUMOylation and SUMO-modified substrate proteins. SUMOylation is critical to cancer stem cell maintenance and self-renewal. Knockdown of SUMO activating enzyme E1 or SUMO conjugating enzyme (E2) inhibits maintenance and self-renewal of colorectal cancer stem cells [30]. The SUMOylated MAFB promotes colorectal cancer tumorigenesis through cell cycle regulation [43]. Similarly, SUMOylation of Akt is required for cell growth and tumorigenesis, and K276 is the major SUMO acceptor site of Akt [44].

### 4.2. SUMOylation and cardiac disease

Recent studies show that protein SUMOylation plays an important role in cardiac function, and balanced SUMOylation/deSUMOylation is important for proper cardiac development, metabolism and stress adaptation [6,45–47]. SUMOylation is attempted to treat cardiac disease. The increase of Ubc9-mediated SUMOylation may represent a novel strategy for increasing autophagic flux and ameliorating morbidity in proteotoxic cardiac disease [47]. The Ubc9/The PML/RNF4 (a SUMO-targeted ubiquitin ligase) axis plays a critical role as an important SUMO pathway in cardiac fibrosis, which provides an attractive therapeutic target for treatment of cardiac fibrosis and heart failure by modulating the signal axis pathway [45].

### 4.3. SUMOylation and neurodegenerative disease

Neurodegenerative diseases often involve the formation of abnormal and toxic protein aggregates, which are thought to be the primary factor in neurodegenerative disease occurrence and progression. Accumulating evidences demonstrate perturbations of neuronal SUMOylation contribute to numerous pathological conditions and neurological disorders [7,48,49].

#### 4.3.1. Huntington's disease

It is known abnormality of HTT protein modification is associated with Huntington's disease (HD) [50]. A pathogenic fragment of HTT can be modified by SUMO-1 at the Lys residue, HTT-fragment SUMOylation increases neurodegeneration in HD model. In addition, HTT SUMOylation increases the degradation of ubiquitin–proteasome pathway, resulting in the accumulation of HTT, which finally leads to HD [32]. Other reports show HTT is modified by SUMO-2 to modulate insoluble mutant HTT protein accumulation, and PIAS1 enhances SUMO-2 modification [50,51].

#### 4.3.2. Parkinson's disease

SUMOylation is linked with the development of Parkinson's disease (PD) [52]. The α-synuclein, which highly expressed in the brain and associated with PD, has been verified to be SUMOylated preferentially by SUMO-1 [53]. The SUMOylation of DJ-1 plays an intriguing potential role for PD. The SUMO-modified DJ-1 participates in the transcriptional regulation of genes concerned with the cellular regulation of oxidative stress. Whereas DJ-1 mutation will prevent SUMOylation and abolish all of its known functions [54,55]. The PIAS family members, as SUMO E3 proteins, interact with DJ-1 and stimulate its SUMOylation in the process of eliminating ROS [52,56].

#### 4.3.3. Alzheimer's disease

Alzheimer's disease (AD) is an age-dependent, progressive neurodegenerative disorder that is characterized by amyloid-β (Aβ) plaque formation [7] and the presence of neurofibrillary tangles composed of hyperphosphorylated tau protein. Previous studies indicated that SUMO-3 overexpression affects Aβ levels [57]. SUMO-1 also modulates Aβ generation via accumulation of the Alzheimer's β-secretase BACE1 [58]. The SUMOylation of tau protein is also associated with the development of AD [59,60]. Tau protein can be both SUMOylated and ubiquitylated [61]. Inhibition of the proteasomal degradation pathway increases the tau ubiquitination and decreases its SUMOylation, suggesting that SUMO and ubiquitin might compete to regulate tau stability [53].

### 4.4. SUMOylation and innate immunity

SUMOylation also involves in the replication of a large number of viruses, either through the direct modification of viral proteins or through the modulation of cellular proteins implicated in antiviral defense. There is growing evidence that SUMO regulates several host proteins involved in intrinsic and innate immunity, thereby contributing to the process governing interferon production during viral infection [62–65]. SUMOylation of proteins have been implicated in the resistance to RNA viral infection. For DNA viruses, SUMOylation promotes the stability of the DNA sensor cGAS and the adaptor STING to regulate the kinetics of response to DNA virus [66,67].

SUMOylation is a novel post-translational modification for TANK-binding kinase 1 (TBK1) [63]. TBK1 kinase activity is required to allow the attachment of SUMO-1 or SUMO-2/3 proteins, and a SUMO modification at K694 contributes to the antiviral function of TBK1, while the viral protein Gam1 antagonizes this post-translational modification. Another study identified SUMO1 was the key gene for inflammatory breast cancer [63]. TRIM38 acts as an E3 ubiquitin or SUMO ligase, which targets key cellular signalling components, regulating the innate immune and inflammatory responses [68]. SUMOylation of NF-κB essential molecule NEMO augments NF-κB activity, NF-κB-dependent cytokine production and pancreatic inflammation [69]. In summary, SUMOylation has been deeply studied recently, and its understanding could be vital for developing potential therapeutic strategies.

## 5. Approaches to identify SUMOylation site

**Figure 3.**
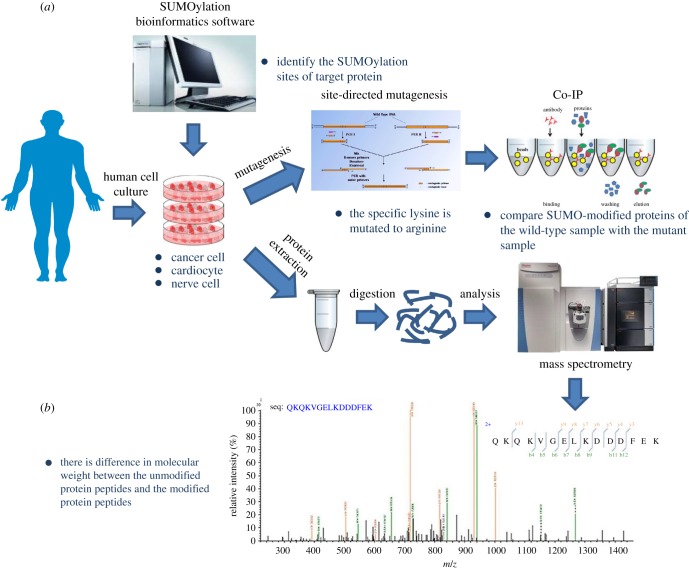
Several methods to identify SUMOylation site. SUMO modification sites are predicted using bioinformatics software analysis. The prediction site is verified using the following methods. (*a*) The SUMOylation site is analysed through site-directed mutagenesis and Co-IP. (*b*) SUMO modification site is identified by mass spectrometry.

Nowadays, the identification of SUMO modification has faced several challenges due to low abundance of most SUMOylated proteins. The approaches for SUMOylation identification mainly include the bioinformatics coupled with the amino acid site-directed mutagenesis and mass spectrometry (MS)-based proteomics analysis. We can predict protein SUMOylation sites by the analogue computation bioinformatics, which is further verified by amino acid site-directed mutagenesis. In addition, the variable SUMO modification sites of target proteins are identified by MS-based techniques and the biochemical validation (figure 3).

The identification of SUMOylation sites and SUMO-interaction motifs in proteins is fundamental for understanding biological functions and regulatory mechanisms of SUMOs. Recently several bioinformatics software tools have been developed to predict SUMOylation modification (table 2), including SUMOsp [70,71], SUMOplot [72], SUMOpre [73], FindSUMO [74], SUMmOn [75], SUMOtr [76], SeeSUMO [77], SUMOhydro [78] and SUMOhunt [79]. The SUMOsp and SUMOplot approaches predict SUMO modification sites mainly based on the conserved sequence ψ–K–X–E/D. Generally these bioinformatics methods focus on the characteristics of a rigorous algorithm of the existing SUMO-specific sites on the substrate proteins, and some even consider the protein space structure and hydrophobicity. These bioinformatics predictions usually need experimental data validation.

**Table 2.** The methods of predicting SUMO modification sites. GPS, group-based prediction system; MotifX, statistical phosphorylation sites prediction method; PSSM, position-specific scoring matrix; SVMa, support vector machines; MS, mass spectrometry. WEKA, Waikato environment of knowledge analysis.

bioinformatic tools	characteristic	year	website	free or not free	refs
SUMOsp	including SUMOsp 1.0 and 2.0 based on two algorithms applied GPS and motifX in SUMOsp	2006	http://sumosp.biocuckoo.org/	free	[70,71]
SUMOplot	a commercially available SUMOylation site predictor based on a SUMO-modified conserved sequence and hydrophobicity analysis	2006	http://www.abgent.com/tools/sumoplot/	free	[72]
SUMOpre	using a probabilistic model for prediction	2008	http://spg.biosci.tsinghua.edu.cn/service/sumoprd/predict.cgi (unable to access)	unknown	[73]
FindSUMO	based on PSSM	2008	http://findingsumo.com.cutestat.com/	not free	[74]
SUMmOn	based on the sequence information automated pattern recognition tool detects PTM fragment ion series within complex MS/MS spectra calculating two independent scores, one for the modification and one for the target peptide	2008	http://summon.sourceforge.net/	free	[75]
SUMOtr	using structure and sequence information higher in correlation coefficient and sensitivity	2010		unknown	[76]
SeeSUMO	using the domain-specific knowledge in terms of relevant biological features for input vector encoding	2011	http://bioinfo.ggc.org/seesumo/ (unable to access)	unknown	[77]
SUMOhydro	based on hydrophobic properties using SVM for classification	2012	http://protein.cau.edu.cn/others/SUMOhydro/	free	[78]
SUMOhunt	using random forest-based classifier provided in WEKA needing sequence and several physico-chemical properties	2013		unknown	[79]

### 5.1. Small ubiquitin-like modifier modification site is identified by mass spectrometry

Despite the powerful SUMO-modified prediction software providing a theoretical basis for the prediction of SUMO modification sites, the precise identification of SUMO modification sites is very important for investigating the target protein functions. Recent advances in MS-based proteomics have greatly facilitated the robust identification and quantification of PTMs [80,81], including SUMO modification. The most common approach is to isolate the target SUMOylated protein by affinity chromatography and to identify by MS [61,82–84]. It is noted that this approach requires the expression of a mutant form of SUMO, in which the residue preceding the C-terminal Gly–Gly (diGly) is replaced with a Lys (SUMO (KGG)) [85]. Digestion of SUMO (KGG) protein conjugates with endoproteinase Lys-C yields a diGly motif attached to target lysines. Peptides containing this adduct are enriched using a diGly-Lys (K-ɛ-GG)-specific antibody and identified by MS. This diGly signature is characteristic of SUMO(KGG) conjugation alone, as no other Ubl yields this adduct upon Lys-C digestion [85].

MS-based identification of SUMOylated sites is hampered by the large peptide remnant of SUMO proteins that are left on the modified Lys residue upon tryptic digestion. Regarding this problem, tandem affinity purification can carry out a more efficient enrichment of SUMOylated proteins by allowing the use of strong denaturing conditions generally to remove most of the contaminant proteins [86].

### 5.2. SUMOylation site is confirmed by site-directed mutagenesis

The Lys site on a protein, possibly modified by the SUMO molecule, is usually mutated to the Arg residue by site-directed mutagenesis to check biological function changes. This classic biochemical method is very efficient to confirm the protein SUMOylation site, but the throughput is not high as MS. For instance, the SUMOylation of TARBP2 at K52 is found to require for regulating miRNA/siRNA efficiency by this biochemical method [87].

### 5.3. Proximity ligation assays for detection protein SUMOylation *in vivo*

Detection of protein SUMOylation *in situ* by proximity ligation assays (PLA) allows easy visualization of endogenous protein–protein interactions at the single molecule level [88–91]. PLA relies on the use of combinations of antibodies coupled to complementary oligonucleotides that are amplified and revealed with a fluorescent probe, with each spot representing a single protein–protein interaction. In PLA, one antibody is directed against the substrate ‘protein X’, while another targets SUMO-1, SUMO-2/3 or ubiquitin. PLA could detect a ‘SUMOylated protein X’ fraction, but also ‘protein X’ interacting with other SUMOylated proteins. PLA offers a quick, cheap and ultrasensitive way for initial testing of ubiquitin-like modifications [92].

### 5.4. *In situ* SUMOylation assay

Another method is *in situ* SUMOylation assay [93,94], which is based on the fluorescence detection of SUMOylation and deSUMOylation in cultured cells. The recombinant green fluorescence protein fused to the SUMO-1 (GFP-tagging SUMO1) is used to visualize the nuclear rim, nucleolus and nuclear bodies. These GFP signals represent cellular regions where SUMOylation efficiently takes place. The recombinant SUMO-specific protease SENP1 catalytic domain is added to erase GFP signals when deSUMOylation happens. Some novel integrative technologies have been developed according to the above principles. A semi-intact cell system, in combination with siRNA-based knockdown of nucleoporin RanBP2 [93], reveals a modulatory role of RanBP2 in the nuclear rim and PML bodies.

## 6. Prospective

SUMO modification has been in existence more than a decade. SUMOs have been established as essential regulators of many cellular functions. It is considered to be one of the important factors regulating the function of the intracellular protein, and abnormal protein SUMOylation will lead to the occurrence of disease.

Recently, the relationship of protein SUMOylation and autophagy has been studied. Autophagy is a catabolic process that facilitates nutrient recycling via degradation of damaged organelles and proteins through lysosomal-mediated degradation [46,95–97]. Autophagy is one of the main mechanisms in the pathophysiology of neurodegenerative disease. The accumulation of autophagic vacuoles (AVs) in affected neurons is responsible for Aβ production. Previous investigation has proved that SUMOylation is associated with autophagy. Overexpression of SUMO1 increased autophagic activation, inducing the formation of LC3-II-positive AVs in neuroglioma H4 cells [98]. Ubc9 overexpression induced relatively high levels of autophagy and led to an increase in autophagic flux, while Ubc9 depletion led to decreased LC3-II expression. This may represent a novel strategy for increasing autophagic flux and ameliorating morbidity in proteotoxic cardiac disease [6]. Conversely, autophagy can regulate Ubc9 levels during viral-mediated tumorigenesis. Ubc9 and autophagy are important co-factors to prime early stages of human papillomavirus-mediated tumorigenesis [99].

With the continuous development of bioinformatics and MS, several accurate and high-throughput methods have been implemented to explore SUMO-modified substrates and sites, which is helpful for deciphering protein SUMOylation-mediated molecular mechanisms of disease.

## Data accessibility

This article has no additional data.
